# No Change in Risk for Antibiotic-Resistant Salmonellosis from Beef, United States, 2002–2010

**DOI:** 10.3201/eid2609.190922

**Published:** 2020-09

**Authors:** Solenne Costard, Jane G. Pouzou, Keith E. Belk, Paul S. Morley, John W. Schmidt, Tommy L. Wheeler, Terrance M. Arthur, Francisco J. Zagmutt

**Affiliations:** EpiX Analytics, Fort Collins, Colorado, USA (S. Costard, J.G. Pouzou, F.J. Zagmutt);; Colorado State University, Fort Collins (K.E. Belk); Texas A&M University, Canyon, Texas, USA (P.S. Morley);; US Department of Agriculture, Clay Center, Nebraska, USA (J.W. Schmidt, T.L. Wheeler, T.M. Arthur)

**Keywords:** Risk assessment, antibacterial drug resistance, antibiotic resistance, antimicrobial resistance, foodborne diseases, nontyphoidal salmonellosis, beef, United States, food safety, bacteria, salmonella

## Abstract

Restricting antibiotic use in food production animals is a target for reducing antimicrobial drug–resistant infections in humans. To estimate the probability of antibiotic-resistant nontyphoidal salmonellosis per meal made with beef during 2002–2010, we used US surveillance data. Applying data for nontyphoidal *Salmonella* in raised-without-antibiotics cattle, we tested the effect of removing antibiotic use from all beef cattle production. We found an average of 1.2 antibiotic**-**resistant nontyphoidal salmonellosis cases per 1 million beef meals made with beef initially contaminated with antibiotic-resistant nontyphoidal *Salmonella* at slaughter or retail and 0.031 cases per 1 million meals irrespective of beef contamination status. Neither outcome showed sustained change except for increases in 2003 and 2009 (>98% confidence) when larger or more outbreaks occurred. Switching all beef production to a raised-without-antibiotics system may not have a significant effect on antibiotic-resistant nontyphoidal salmonellosis (94.3% confidence).

Increased antimicrobial resistance (AMR), or antibiotic resistance, has resulted in initiatives to reduce the use of antibiotics in food production animals (*1*,*2*), but quantification of the public health effects of decreasing antibiotic use in livestock remains limited (*3*,*4*). Reduction of antibiotic use in livestock can lower resistance prevalence (i.e., proportion of pathogens with resistance) in animals (*4*), but some studies show that pathogen prevalence may be higher in livestock raised without antibiotics (*5*). Because transmission of foodborne pathogens is proportional to the prevalence of pathogens in the food source (*6*), quantifying the change in human antibiotic-resistant foodborne illnesses resulting from reduced antibiotic use in livestock is vital.

In the United States, the most common bacterial cause of foodborne illness is nontyphoidal *Salmonella* (NTS), which leads to >1 million foodborne illnesses and 20,000 hospitalizations per year (*7*). Antibiotic-resistant NTS is among the top 18 AMR threats in the United States (*8*), causing 100,000 infections annually. The Centers for Disease Control and Prevention National Antimicrobial Resistance Monitoring System (NARMS) tracks resistance to 25 antibiotics in patient samples positive for isolates such as NTS (*9*), including the clinically relevant antibiotics ciprofloxacin and ceftriaxone.

Multiple assessments of human AMR risk from meats have been performed (*10*–*14*). However, most focused on only 1 class of antibiotic (*10*,*11*), had limited or no longitudinal data (*14*), or were not based on nationwide surveillance at the animal source (*11*). Quantitative assessments of AMR risk with a more comprehensive resistance definition (*15*), such as resistance to any class, or to >3 classes, that use representative, longitudinal data, are critical to defining the risks and benefits from policy with regard to antibiotic use in livestock (*3*). Surveillance studies of antibiotic use and AMR in humans and livestock can be used to generate estimates of risk based on empirical data and can show the results of long-term conditions or systematic changes over time.

Our objective with this study was to use beef as a model to quantify trends in the longitudinal relationship human NTS infections and antibiotic-resistant NTS in meats. We also used the estimates to predict change in antibiotic-resistant salmonellosis resulting from hypothetical scenarios of antibiotic restriction in beef cattle.

## Methods

We developed a stochastic model to quantify the risk for antibiotic-resistant nontyphoidal salmonellosis per meal made with beef during 2002–2010. Our model follows the method of previously published AMR risk assessments (*6*,*16*) but uniquely addresses temporal changes and relies solely on nationwide surveillance data (Appendix Table 1).

**Figure 1:**
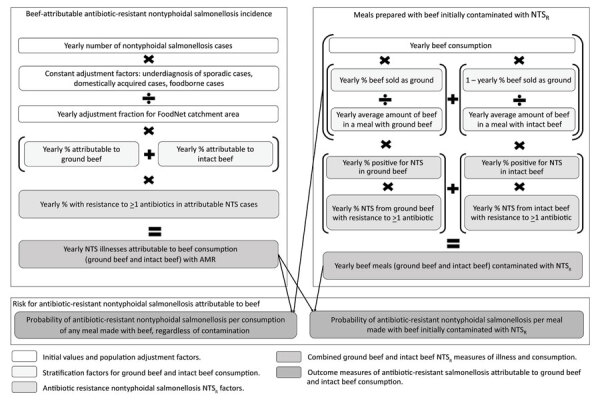
Conceptual model and data sources for calculation of risk for beef-attributable antibiotic-resistant nontyphoidal salmonellosis per 1 million beef meals (*P_ill_*) in study of risk for antimicrobial-resistant salmonellosis from beef, United States, 2002–2010. NTS, nontyphoidal *Salmonella*; NTS_r_, antibiotic-resistant NTS.

**Figure 2:**
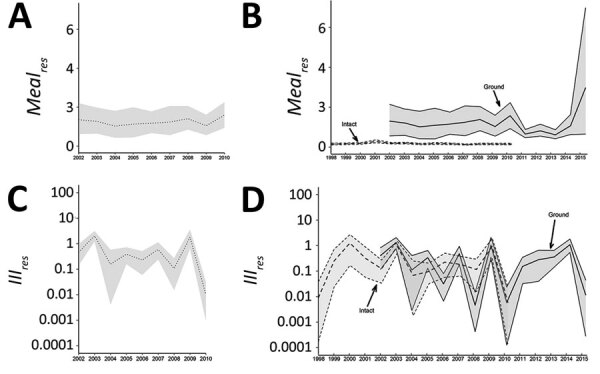
Estimates of the number of annual beef meals (in millions) prepared with beef initially contaminated with NTS resistant to >1 antibiotic (*Meal_res_*) and of the incidence of salmonellosis with resistance to >1 antibiotic and attributable to beef (*Ill_res_*) per 100,000 persons, United States, 2002–2010. A) *Meal_res_* for total beef, 2002–2010. B) *Meal_res_* stratified as ground (2002–2014) or intact (1998–2010) cuts. C) *Ill_res_*, 2002–2010. D) *Ill_res_* attributable to beef stratified as ground (2002–2014) or intact (1998–2010) cuts. Center lines represent means; gray shading represents 95% credible intervals; for panels B and D, light gray shading represents intact beef and dark gray shading indicates ground beef.

We used this model for 3 objectives: 1) estimate the risk for antibiotic-resistant nontyphoidal salmonellosis per meal made with beef, using the yearly cases of illnesses (*Ill_res_*) and the number of meals made with beef that year (*Meal_res_*) (Figure 1); 2) evaluate change over time in all model outcomes; and 3) using national surveillance data, assess the effect that potential future restrictions on antibiotic use in beef cattle would have on antibiotic-resistant nontyphoidal salmonellosis disease burden (Appendix).

### Risk for Antibiotic-Resistant Nontyphoidal Salmonellosis Attributable to Beef

#### Annual Incidence of Beef-Attributable Antibiotic-Resistant Nontyphoidal Salmonellosis (*Ill_res_*) per 100,000 persons

We obtained the annual total of nontyphoidal salmonellosis cases in the United States for 1998–2015 from FoodNet (https://www.cdc.gov/foodnet/index.html), an active foodborne disease surveillance system, after adjusting for the proportion of the US population included in FoodNet surveillance sites. To correct for underdiagnosis and restrict case estimates to domestically acquired foodborne cases, we also included adjustment factors constant for the study period. By using annual food attribution estimates derived from the National Outbreak Reporting System (NORS; https://www.cdc.gov/nors/index.html), cases of nontyphoidal salmonellosis were further restricted to foodborne cases attributed to ground beef and intact beef. To ensure that the resistance fraction is specific to nontyphoidal salmonellosis attributed to consumption of beef, we estimated the fraction of beef-attributed nontyphoidal salmonellosis cases with AMR by matching cases in the Centers for Disease Control and Prevention data collected from clinical patient samples as part of NARMS (*17*) with beef-attributable outbreak data from NORS by using sample metadata, (Appendix Table 1). For the purpose of trend tests for this variable, we calculated incidence of *Ill_res_* by using the population of the United States in the relevant year.

**Table 1:** Risk calculation inputs and outputs for study of risk for antimicrobial-resistant salmonellosis from beef, United States, 2002–2010*

Input	**Mean (95% CrI)**	
	2002–2010†	Any years with data
Meals prepared with beef		
Total	554B (527B–581B)	554B (527B–581B)
Ground	326B (306B–345B)	497B (473B–521B)
Intact	228B (219B–238B)	329B (319B–339B)
Meals prepared with beef carrying NTS		
Total	24.9B (22.5B–27.0B)	24.9B (22.5B–27.0B)
Ground	22.5B (20.3B–24.9B)	36.3B (31.1B–42.7B)
Intact	2.4B (2.1B–2.7B)	4.2B (3.7B–4.6B)
Meals prepared with beef carrying NTS_R_ (*Meal_res_*)		
Total	11.2B (9.08B–13.54B)	11.2B (9.08B–13.54B)
Ground	10.4B (8.3B–12.71B)	16.22B (12.69B–20.90B)
Intact	811M (708M–925M)	1.30B (1.16B–1.46B)
Nontyphoidal salmonellosis attributable to beef, no. cases/100,000 US population		
Total	15.10 (0.096–44.44)	15.10 (0.096–44.44)
Ground	8.27 (0.028–25.99)	8.99 (0.028–26.94)
Intact	6.83 (0.028–20.07)	6.75 (0.043–19.72)
*Ill_res_/*100,000 US population		
Total	0.64 (0.0036,2.75)	0.64 (0.0036–2.75)
Ground	0.39 (0.0007,1.54)	0.36 (0.0008–1.46)
Intact	0.25 (0.001,1.25)	0.31 (0.00084–1.54)
Nontyphoidal salmonellosis attributable to beef/1 million beef meals		
Total	0.74 (0.0046–2.20)	0.74 (0.0046–2.20)
Ground	0.70 (0.0022–2.25)	0.78 (0.0024–2.35)
Intact	0.81 (0.0034–2.38)	0.78 (0.0051–2.29)
Nontyphoidal salmonellosis attributable to beef/1 million NTS beef meals		
Total	17.1 (11.4–24.0)	17.1 (11.4–24.0)
Ground	10.2 (6.73–14,4)	12.9 (8.6–18.2)
Intact	82.1 (53.8–118.1)	70.4 (46.7–100)
*P_mea_*_l_		
Total	0.031 (0.00018–0.14)	0.031 (0.00018–0.14)
Ground	0.031(0.000056–0.13)	0.031 (0.000067–0.13)
Intact	0.032 (0.0001–0.15)	0.036 (0.00013–0.18)
*P_ill_*		
Total	1.78 (0.007–8.56)	1.78 (0.007–8.56)
Ground	1.15 (0.001–5.38)	1.25 (0.001–5.21)
Intact	9.10 (0.039–47.21)	9.48 (0.032–50.19)

*Years included are 2002–2015 for ground beef, 1998–2010 for intact beef, and 2002–2010 for total beef. Calculations include measures of exposure (meals prepared from beef with various states of microbiological contamination), disease incidence (no. illness cases/100,000 US population), and different measures of disease risk per meals consumed. B, billion; *Ill_res_, *antibiotic-resistant nontyphoidal salmonellosis attributable to beef; M, million; NTS, nontyphoidal *Salmonella*; NTS_R_, antibiotic-resistant NTS; *P_meal_*, antibiotic-resistant nontyphoidal salmonellosis/1 million beef meals; *P_ill_*, antibiotic-resistant nontyphoidal salmonellosis/1 million NTS_R_ beef meals. †Years 2002–2010 summary statistics reflect the data used to create the combined totals.

#### Annual Meals Prepared with Beef Initially Contaminated with Antibiotic-Resistant NTS (*Meals_res_*)

We calculated the number of beef meals consumed annually in the United States by using beef disappearance data from the US Department of Agriculture (USDA) (*18*) and the mean grams of beef consumed per beef meal from the National Health and Nutrition Examination Survey (*19*). We estimated the prevalence of NTS in beef by using USDA Food Safety and Inspection Service surveillance data, and we derived the fraction of isolates with AMR from USDA NARMS and US Food and Drug Administration NARMS data (*9*). *Meals_res_* were stratified by beef cut (ground beef data for 2002–2015 vs. intact beef for 1998–2010). By using *Meals_res_*, we assumed that the beef used to prepare a meal was initially contaminated (as measured at the slaughter plant or retail) with the pathogen. This assumption does not necessarily mean that the actual meal consumed was contaminated because safe cooking and handling practices would reduce or completely inactivate the bacterial load.

#### Risk for Antibiotic-Resistant Nontyphoidal Salmonellosis per Beef Meal 

Dividing *Ill_res_* by *Meals_res_* resulted in the probability of antibiotic-resistant nontyphoidal salmonellosis per meal made with beef initially contaminated with antibiotic-resistant NTS (*P_ill_*). Also, by using all meals in the denominator, we calculated the probability of antibiotic-resistant nontyphoidal salmonellosis per meal made with beef, regardless of contamination status (*P_meal_*) (Figure 1). We report both risk outcomes per 1 million meals, on a per-year basis (*P_ill_* and *P_meal_*) and as the mean of each for all years combined (*P_ill,overall_* and *P_meal,overall_*). We repeated the analyses for NTS with multidrug resistance (NTS_MDR_) (i.e., resistance to >3 antimicrobial classes) and for clinically relevant resistance (NTS_CRR_), also known as resistance of concern (i.e., resistance to >5 drugs or quinolones [ciprofloxacin] or third-generation cephalosporins [ceftriaxone]) (*8*).

### Testing for Temporal Changes

To identify the confidence of a consistent increase (or decrease) in each outcome over the study period, we used Mann-Kendall trend test bootstrapping (*20*). In addition, used numerical integration to compute the confidence in pairwise year-to-year Bayesian posterior differences (*21*) and the difference between the mean of each outcome in the past 5 years versus the remaining previous years. Unlike the Mann-Kendall tests, the year-to-year test identified short-term changes, and the comparison of the first versus the last years of the study period provided an assessment of nonconsistent changes during the study period.

### Scenario Analysis: Effects of Hypothetical Antibiotic Restriction in Beef Production

#### Relationship between Antibiotic Use and Antibiotic-Resistant NTS in Beef

To model the relationship between antibiotic use and antibiotic-resistant NTS, we used nationwide data (C.P. Fossler, USDA, pers. comm., 2018 Jul 16) from the National Animal Health Monitoring System feedlot survey (*23*). The feedlot survey is based on a nationwide representative sample of farms and thus captures the effect of long-term and current antibiotic practices on AMR. In the survey, individual fecal pats from raised-without-antibiotics cattle and conventionally raised cattle were collected to estimate the prevalence of NTS isolates and the fraction of these with AMR. These 2 parameters were combined to measure the overall prevalence of antibiotic-resistant NTS in raised-without-antibiotics cattle and conventionally raised cattle and to derive the relative risk (RR) of antibiotic-resistant NTS prevalence in raised-without-antibiotics versus conventionally raised cattle.

#### Prediction of Changes in Beef-Attributable Antibiotic-Resistant Nontyphoidal Salmonellosis

We constructed 2 scenarios to evaluate *Ill_res_* changes from hypothetical antibiotic restriction in beef production. We assumed no changes in slaughtering, processing, consumer habits, and food preparation. 

For scenario 1, we estimated the change in antibiotic-resistant nontyphoidal salmonellosis if all beef production were switched to raised-without-antibiotics by using the annual estimated *Ill_res_* for 2002–2010 and the RR of antibiotic-resistant NTS prevalence in raised-without-antibiotics versus conventionally raised cattle. By doing so, we assumed that the animal-level prevalence of antibiotic-resistant NTS is proportional (but not equal to) its prevalence in meals prepared with beef and that RR has a direct linear effect on the change in *Ill_res_*. This relationship is documented for food pathogens (*6*,*24*), including NTS (*25*), so here we assumed that it extends to antibiotic-resistant isolates. 

To relax this assumption, for scenario 2, we empirically estimated the relationship between antibiotic-resistant NTS prevalence in beef and *Ill_res_* via Poisson regression and used the Poisson regression to create an adjustment factor to the calculations done for scenario 1. For each scenario, we reported the posterior confidence in the change in *Ill_res_* being <0 (i.e., reduction of antibiotic-resistant nontyphoidal salmonellosis) for each year of the study and for all years combined.

### Model Implementation

We performed all analyses by using R version 3.4.1 (https://www.R-project.org). We used Monte Carlo simulation to calculate the posterior uncertainty in all outcomes. Statistical significance was assessed at the 95% confidence level. We report mean and 95% credible interval (CrI) for all outcomes. We performed a sensitivity analysis of the key drivers of *P_ill_* and *P_ill,overall_* by calculating the effect that extreme values of each input had on the output means (Appendix).

## Results

### Descriptive Statistics of Main Parameters and Risk Measures

During 2002–2010, approximately 554 beef meals were consumed, 59% as ground beef. Of these meals, 4% came from beef at slaughter or retail with NTS, half of which were antibiotic-resistant (total *Meals_res_* 2002–2010 = 11.23 billion, 95% CrI 9.08–13.54 billion). Approximately 93% of meals with beef initially contaminated with antibiotic-resistant NTS (10.4 billion meals, 95% CrI 8.3–12.73 billion) were made with ground beef (Figure 2), resulting from higher prevalence of both NTS and antibiotic-resistant NTS in ground than intact beef (Table 1). Yet, the attribution of nontyphoidal salmonellosis, regardless whether antibiotic resistance, was relatively even between ground and intact beef (Figure 2). The total incidence of *Ill_res_* was 0.64 (0.0036–2.75)/100,000 persons.

During 2002–2010, the mean risk for antibiotic-resistant nontyphoidal salmonellosis was 0.031 cases (95% CrI 0.00018–0.14)/1 million beef meals; intact and ground beef contributed equally to the rate (Table 1; Figure 2). The risk per million beef meals initially contaminated with NTS was 1.8 (95% CrI 0.007–8.5) overall, 1.16 (95% CrI 0.0015–5.2) for ground beef and 9.5 (95% CrI 0.03–50) for intact beef (Figure 2). The higher *P_ill,overall_* for intact beef possibly indicates a higher risk from consumption of intact beef carrying antibiotic-resistant NTS.

### Tests for Temporal Changes in Main Parameters and Risk Measures

**Figure 3:**
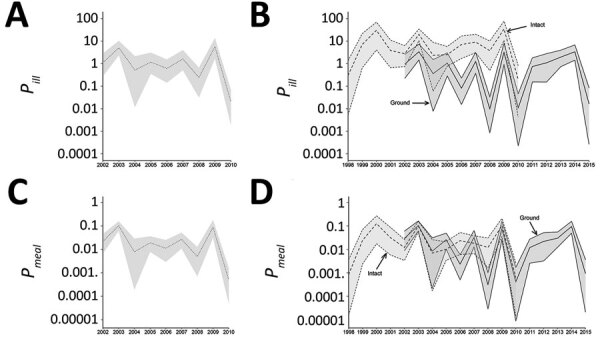
Estimates of *Meal_res_* and *Ill_res_*, United States, 2002–2010. A) *Meal_res_* for total beef, 2002–2010. B) *Meal_res_* stratified as ground (2002–2014) or intact (1998–2010) cuts. C) *Ill_res_*, 2002–2010. D) *Ill_res_* attributable to beef stratified as ground (2002–2014) or intact (1998–2010) cuts. Center lines represent means; gray shading represents 95% credible intervals; for panels B and D, light gray shading represents intact beef and dark gray shading indicates ground beef. *Ill_res_*, incidence (per 100,000 population) of salmonellosis with resistance to >1 antibiotic and attributable to beef; *Meal_res_*, number of annual meals prepared with beef initially contaminated with NTS resistant to >1 antibiotic; NTS, nontyphoidal *Salmonella*.

None of the tested parameters or outcomes based on a resistance definition of >1 antibiotic (i.e., *Meals_res_* or *Ill_res_* per 100,000 population [Figure 2], or *P_ill_* or *P_meal_* [Figure 3]) showed a sustained change (Table 2). We also observed no change when we used multidrug resistance (MDR) and clinically relevant resistance (CRR) as the definition of resistance (Table 2; Appendix Figures 5–8), except that meals made with ground beef contaminated with NTS_CRR_ declined during 2002–2015. More differences based on the past 5 years were found. The risk for NTS_MDR_ per 1 million meals made with ground beef initially contaminated with NTS_MDR_ increased during 2010–2015, while the number of these meals made with NTS_MDR_-contaminated ground beef decreased (Table 2). In contrast, for CRR, the beef-attributable risk for CRR nontyphoidal salmonellosis was significantly lower for all beef meals initially contaminated—and ground beef specifically—in the last 5 years of data, as were both the incidence of CRR nontyphoidal salmonellosis and its risk per 1 million beef meals, overall and for intact beef (Table 2).

**Table 2:** Confidence in a significant monotonic trend in the data (bootstrapped Mann-Kendall test) and in the difference between posteriors estimates of the last 5 years versus the previous years for measures of beef consumption, NTS illnesses, and risk for antimicrobial resistant salmonellosis from beef, United States*

Variable	*Monotonic (confidence trend exists), %*	*Last 5 vs. previous years (confidence difference exists), %*	*Years found significantly higher based on all pairwise comparisons†*
*Meals_res_*	38.2	68.7	None
Ground	66.6	44.7	None
Intact	88.0	93.8	None
*Meals_res,MDR_*	87.0	86.3	None
Ground	94.5 (D)	85.8	None
Intact	53.0	98.4 (D)	2001
*Meals_res,CRR_*	82.0	85.7	None
Ground	96.7 (D)	94.5	None
Intact	34.2	91.4	None
*Ill_res_*	82.0	67.2	2003, 2009
Ground	66.8	55.3	2003, 2009, 2014
Intact	57.3	69.0	2003, 2009
*Ill_res,MDR_*	86.6	87.2	2003
Ground	67.0	57.6	2003, 2014
Intact	61.9	84.5	2000, 2003
*Ill_res,CRR_*	90.6	100 (D)	2003
Ground	70.1	54.7	2003
Intact	66.2	98.6 (D)	2000, 2003
*P_meal_*	82.1	84.7	2003, 2009
Ground	62.9	54.7	2003, 2009, 2014
Intact	56.8	70.5	2003, 2009
*P_meal, MDR_*	86.7	97.7 (D)	2003
Ground	66.9	50.8	2003, 2014
Intact	61.9	85.0	2000, 2003
*P_meal, CRR_*	91.0	99.9 (D)	2003
Ground	67.2	49.9	2003
Intact	70.4	98.7(D)	2003
*P_ill_*	87.3	84.1	2003, 2009
Ground	54.4	75.2	2003, 2009, 2014
Intact	42.6	49.9	2003, 2009
*P_ill,MDR_*	82.2	86.0	2003
Ground	46.5	97.6 (I)	2003, 2014
Intact	53.2	66.1	2003
*P_ill,CRR_*	87.0	99.6 (D)	2003
Ground	36.8	99.9 (D)	2003, 2014
Intact	70.5	91.7	2000, 2003

*D indicates that a significant decrease was found; I indicates that a significant increase was found, based on a 95% limit. CRR, clinically relevant resistance; *Ill_res_*, human cases of beef-attributable antibiotic-resistant NTS; *Ill_res,MDR_*, human cases of beef-attributable MDR NTS; *Ill_res,CRR_*, human cases of beef-attributable CRR NTS; *Meals_res_*, meals prepared with beef initially contaminated with antibiotic-resistant NTS resistant to >1 antibiotic; *Meals_res,MDR_*, meals prepared with beef initially contaminated with NTS resistant to >2 antibiotics; *Meals_res,CRR_*, meals prepared with beef initially contaminated with NTS with CRR; MDR, multidrug-resistant; NTS, nontyphoidal *Salmonella*; *P_meal_*, probability of antibiotic-resistant NTS per meal made with beef of any kind; *P_meal,MDR_*, probability of MDR NTS per meal made with beef of any kind; *P_meal,CRR_*, probability of clinically relevant antibiotic-resistant NTS per meal made of beef of any kind; *P_ill_*, probability of antibiotic-resistant NTS per meal made with beef initially contaminated with antibiotic-resistant NTS; *P_ill,MDR_*, probability of MDR NTS per meal made with beef initially contaminated with MDR NTS; *P_ill,CRR_*, probability of CRR NTS per meal made with beef initially contaminated with CRR NTS.  †Based on pairwise posterior comparisons between all years.

We found some year-to-year variations in *Ill_res_*, *P_ill_*, and *P_meal_* but generally no yearly changes in meals made with beef initially contaminated with antibiotic-resistant NTS (*Meal_res_*)_._ For all beef and for ground beef and intact beef individually, defining resistance as resistance to >1 antibiotic, *Ill_res_*, *P_ill_*, and *P_meal_* were higher in 2003 and 2009 and a peak for ground beef also occurred in 2014. *Meals_res_* showed no significant year-to-year changes for all beef cuts combined. Intact beef *Meals_res_* had 1 peak in 2001 (100% confidence). When MDR and CRR were used as the resistance definition, only the peaks in 2003 and in 2014 remained significant. A peak in some intact beef risks and illnesses was also observed in 2000 (Table 2).

### Scenario Analysis of Changes in Antibiotic-Resistant Nontyphoidal Salmonellosis Resulting from Antibiotic Restriction in Beef Production

**Figure 4:**
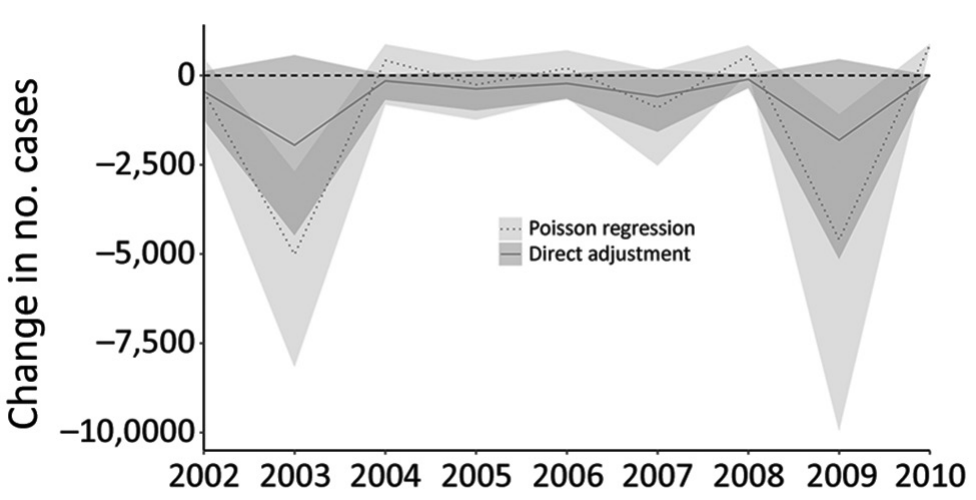
Predicted changes in each year’s cases of antimicrobial-resistant salmonellosis from beef, United States, 2002–2010. Mean and 95% credible intervals of the predicted change are shown for the hypothetical scenario of 100% raised-without-antibiotics beef consumption, assuming a direct linear relationship between prevalence of antimicrobial-resistant *Salmonella* in beef and antimicrobial-resistant salmonellosis cases (solid line and dark grey shading), contrasted with the result from adjusting the relationship of beef resistance and prevalence with human cases based on the Poisson regression between the 2 variables (dotted line and light grey shading).

In the first scenario analysis, we found no significant changes (<94.3% confidence) in antibiotic-resistant salmonellosis for any year when switching from current antibiotic practices to hypothetical 100% raised-without-antibiotics production. The mean change in the number of antibiotic-resistant nontyphoidal salmonellosis cases across the study period was −5,218 (Figure 4), ranging from an additional 1,441 resistant nontyphoidal salmonellosis cases to a reduction of 14,350 cases.

The second scenario (Figure 4), in which the direct linear assumption was relaxed, predicted significant decreases (>98% confidence) in cases for 2003 (−5,152) and 2009 (−4,763) and a significant increase of 1,098 cases (99.9% confidence) in 2010. However, switching to 100% raised-without-antibiotics production did not significantly change the number of antibiotic-resistant nontyphoidal salmonellosis cases over the full study period combining all 9 years (mean −8,588, 95% CrI −27,842 to 16,317, 60% confidence).

## Discussion

Our risk analysis uses nationwide surveillance data on animal production and human illnesses to longitudinally estimate antibiotic-resistant nontyphoidal salmonellosis in the United States and assess how it might be affected by antibiotic restriction in livestock. Our approach is grounded in empirical data and minimizes assumptions while modeling parameter uncertainty and its effect on the results. Although farm-to-fork AMR risk analyses have been published (*10*), recent work has followed more parsimonious approaches like ours (*11*–*14*). However, direct comparison with other published risk analyses is difficult because most focus on the association between antibiotic use and AMR for a single drug and rarely include longitudinal data.

In our 2002–2010 analysis, the risks were stable over time; on average, a case of antibiotic-resistant salmonellosis occurred <1 time per 32 million meals made with beef or <1 time per 500,000 meals made with beef initially contaminated with antibiotic-resistant NTS. Likewise, prevalence of the antibiotic-resistant pathogen in beef available at retail in the United States and in the food production chain remained stable. Exceptions were 2 years in which more beef-attributable illnesses occurred than was typical for other years: 5 average-sized outbreaks (8% of attributable outbreaks) in 2003 and 2 *Salmonella* Montevideo outbreaks with high total case numbers in 2009.

The proportion of MDR and CRR was higher in NTS isolates from NARMS matched to outbreaks in 2003 and 2009 than in other years: 80% of matched samples in 2003 had CRR, and all 2009 *Salmonella* Montevideo matched samples (71% of all matched 2009 cases) had MDR. This increase remained after we adjusted for exposure to infection in the form of meals prepared with beef with NTS and the fraction of these with AMR, which were stable. The association between MDR and CRR and larger/more frequent outbreaks may suggest a link between multidrug resistance and pathogenicity or infectivity, as described by Guillard et al. (*27*). Yet, in vitro phenotypic resistance does not fully capture actual clinical outcomes. Current foodborne surveillance programs do not record outcomes of AMR illnesses such as treatment failures and their consequences (e.g., extra hospitalizations). Estimating treatment failures resulting from resistant infections and the relative contribution of different sources of AMR—including antibiotic use in livestock—would better quantify the societal cost benefit of curtailing resistant illnesses from livestock.

In our analysis, we had to estimate AMR specific to beef-attributable cases because the NARMS database contains salmonellosis cases of any source and yet resistance of salmonella varies by source (*9*). Lacking direct links between the NORS outbreak data used in source attribution and the outbreaks in NARMS, we used timing of the infection, state, and serotype to match cases. Although this method enabled us to approximate resistance in beef-attributable cases (5% vs. 22% AMR across human NARMS samples for NTS over the study period), use of this method probably resulted in some misclassification of the NARMS samples. This issue would be easily alleviated if a unique outbreak identifier were available in both datasets.

Of note, the per-portion risk for susceptible or resistant salmonellosis from beef initially contaminated was »8 times higher for intact cuts of beef than for ground beef. Because the prevalence of susceptible and resistant pathogens is greater for ground beef, the total illnesses are evenly split between types of beef, as are attributed illnesses, a result also noted by Laufer et al. (*28*). Intact cuts include some high-risk foods such as delicatessen roast beef and ready-to-eat products (*29*). Doneness might also partly explain this finding. A survey found that 61% of US consumers preferred their steak medium or rarer (*30*), and another study found that 21% of restaurant customers requested medium or rarer hamburgers (*31*).

Using NTS in beef, beef-attributable salmonellosis cases, and resistance to >1 antibiotic provided a case definition that maximizes the chances of finding a statistical signal in this dataset, should a trend exist in the outcomes. Consequently, the lack of sustained change suggests that the modeled risks were indeed stable nationwide. Assuming that is often described antibiotic use in beef production is a key driver of AMR illnesses in humans, we consider 2 alternative explanations for this stability: either antibiotic use was stable during the study period or sustained use in beef resulted in a plateau in AMR salmonellosis so that changes in use can no longer affect the outcome. Although nationwide data on antibiotic use is unavailable for the study period, antibiotic use in beef is unlikely to have remained stable. For example, the fraction of beef cattle treated with tylosin in feed or water increased from 42.3% in 1999 to 71.2% in 2010 (*32*,*33*), whereas in Canada, where beef production practices are equivalent to those in the United States, overall use in beef decreased during 2008–2012 (*34*). A hypothetical resistance plateau cannot be empirically answered without detailed use data, but its implication is that changes such as the recent US Food and Drug Administration feed directive should eventually reduce beef-attributable antibiotic-resistant nontyphoidal salmonellosis. This hypothesis warrants a re-estimation of our model in the future.

An alternative hypothesis for the lack of change is that antibiotic use in beef does not significantly affect incidence of human AMR salmonellosis. This hypothesis does not necessarily imply a lack of risk but a risk that is too small or confounded to be measured. Empirical data for this effect are scarce because field studies typically link antibiotic use to AMR in animals or animal products, not in human illnesses. Benedict et al. (*35*) described how exposure to antibiotics in feedlot cattle did not affect AMR presence in non–type-specific *Escherichia coli*. Others have described a lower prevalence of resistance resulting from decreased use (*4*), although pathogen prevalence among raised-without-antibiotics livestock may be higher than that among conventionally raised animals (*5*). Although our study cannot confirm or refute this hypothesis, it provides new empirical evidence based on nationwide estimates and can be further updated as antibiotic practices in livestock are documented.

The scenarios with all raised-without-antibiotics beef cattle enabled us to model a hypothetical upper limit of the human health effect of antibiotic reduction and resulted in nonsignificant changes in resistant illnesses overall. This finding held true even under an unrealistic assumption of a direct decrease in resistant illnesses resulting from decreased pathogen prevalence and resistance after complete withdrawal of antibiotics. Being based solely on nationwide estimates—resistant illnesses based on surveillance data and the effect of antibiotic use on antibiotic-resistant NTS based on a nationwide survey (*23*)—these findings suggest that, according to collected surveillance data, reducing antibiotic use in cattle may not significantly reduce antibiotic-resistant nontyphoidal salmonellosis by a measurable level**.** Although external validation is not feasible because no other study, to our knowledge, has directly tested human and animal resistance at a national level, these results are consistent with those of recent studies of cecal contents of fed cattle (*5*) and ground beef (*36*) that found few AMR differences between raised-without-antibiotics and conventionally raised cattle production. Our findings also demonstrate that a direct relationship between prevalence of antibiotic-resistant NTS in beef and resulting AMR salmonellosis is not supported by current surveillance data.

This analysis suggests that the risk of contracting antibiotic-resistant nontyphoidal salmonellosis from beef consumption is <1 time/32 million beef meals and remained stable during 2002–2010. Despite assessing salmonellosis only, our work highlights improvements needed to better quantify the effect that antibiotic use in livestock has on human health: monitoring of clinical outcomes in foodborne surveillance programs, better connection between surveillance for foodborne pathogen resistance and outbreak sourcing, and detailed studies exploring the effect of raised-without-antibiotics production practices on pathogen prevalence and resistance throughout the farm-to-fork production chain. Elucidating not only consumers’ exposure to resistant pathogens but also how exposure translates into resistant illnesses and, ultimately, treatment failures, is required for the development of optimal AMR reduction strategies.

Appendix Supplemental methods and results for study of risk for antibiotic-resistant salmonellosis from beef, United States, 2002–2010.
