# Supplementary material for: No Change in Risk for Antibiotic-Resistant Salmonellosis from Beef, United States, 2002–2010
Source: Emerg Infect Dis. 2020 Sep;26(9):2108–17. doi: 10.3201/eid2609.190922 (PMC7454111; doi:10.3201/eid2609.190922)
Supplement: Appendix — Supplemental methods and results for study of risk for antibiotic-resistant salmonellosis from beef, United States, 2002–2010. [file 19-0922-Techapp-s1.pdf]

Article DOI: https://doi.org/10.3201/eid2609.190922

No Change in Risk for Antibiotic-Resistant Salmonellosis from Beef, 2002–2010

Appendix

Supplemental Description of Methods

We developed a stochastic model to 1) estimate the risk of human antibiotic-resistant non-typhoidal salmonellosis per meal made with beef using the yearly incidence of antibiotic-resistant non-typhoidal salmonellosis illness and number of meals made with beef that year, 2) evaluate temporal trends in all model outcomes over the period 2002-2010, and 3) assess the effect that potential future antibiotic use (AMU) restrictions in beef cattle would have on this antibiotic-resistant non-typhoidal salmonella disease burden, using national surveillance data.

The Appendix Table provides a detailed summary of the variables and sources of data used to estimate each of the outcomes described below.

1. Risk of human antibiotic-resistant non-typhoidal salmonellosis illness attributable to beef

a. Annual incidence of beef-attributable antibiotic-resistant non-typhoidal salmonellosis illness per 100,000 people (Ill_{res})

We estimated the number of nontyphoidal salmonellosis illnesses attributable to beef consumption per year (*Ill*), and the number of these with AMR (Ill_{res}):

$Ill = NTSc \times FB \times UD \times (GBA + IBA) \times DA \div USFN$ [Equation 1]

$Ill_{res} = Ill \times AMR_{perc}$ [Equation 2]

The annual total NTS_c illnesses in the USA from years 1998-2015 were obtained from the FoodNet active surveillance system, and adjusted for the FoodNet catchment area (*USFN*), domestically-acquired fraction (*DA*), underdiagnosis (*UD*), attribution to food (*FB*), and attribution of foodborne cases to ground beef (GBA) or Intact beef (IBA). The adjustment factors *UD*, *FB* and *DA* were constant for the study period.

To derive the AMR fraction specific to beef-attributable cases of human illness, AMR_{perc}, we used metadata available in both datasets (serotype, date, and location) to match cases in the NARMS data collected by the CDC from clinical patient samples (*1*) with outbreaks from NORS attributed to beef consumption (*2*). The NORS data includes information on identified food source, and these variables were used to identify ground vs intact beef-attributable outbreaks among all salmonella outbreaks.

b. Annual meals prepared with beef initially contaminated with antibiotic-resistant non-typhoidal salmonellosis ($Meals_{res}$)

$Meals_{res}$ quantifies the meals initially contaminated (as measured at the slaughter plant or retail) with the pathogen. This doesn't necessarily mean that the actual meal consumed was contaminated, as safe cooking and handling practices would reduce or completely inactivate the bacterial load. $Meals_{res}$ was calculated as the sum of meals prepared with either ground or intact beef initially contaminated with antibiotic-resistant non-typhoidal salmonellosis and consumed annually in the US:

$$Meals_{res} = \frac{(1-GBF)\times Beef_{dtot}}{MS_{IB}} \times Pp_{IB} \times AMR_{IB} + \frac{GBF\times Beef_{dtot}}{MS_{GB}} \times Pp_{GB} \times AMR_{GB} \text{ [Equation 3]}$$

To estimate the annual number of meals, we combined beef disappearance data from USDA Economic Research Service to estimate total beef available for consumption ($Beef_{dtot}$) with the mean amount of beef consumed per meal containing beef stratified by beef cut (MS_{GB} and MS_{IB}) estimated using NHANES data (GB, years 2002-2015 vs IB, years 1998-2010) using the proportion of beef sold as ground beef (National Cattlemen's Beef Association, pers. com., 2018).

We estimated the prevalence *Pp* of nontyphoidal salmonella in beef using samples collected by USDA Food Safety and Inspection Service (FSIS). We then calculated the prevalence of the pathogen with AMR in IB (AMR_{IB} and AMR_{GB}) nontyphoidal salmonella using a combination of USDA-NARMS data from meat samples collected during IB and GB production for the years available and FDA-NARMS retail studies from GB (*3*) for years 2002-2010. Although these datasets are based on a national catchment area and are the most comprehensive sampling efforts to date, the evolution of the FSIS program targets over time and the small sample size of the FDA-NARMS study in particular may limit the ability to calculate true prevalence from these data.

c. Risk of antibiotic-resistant non-typhoidal salmonellosis per meal with beef

We estimated the probability of human antibiotic-resistant non-typhoidal salmonellosis illness per meal made with beef initially contaminated with antibiotic-resistant non-typhoidal salmonellosis (P_{ill}) by dividing the estimated number of antibiotic-resistant non-typhoidal salmonellosis illnesses for a given year (Ill_{res}) by the number of meals made with beef with antibiotic-resistant non-typhoidal salmonellosis that year ($Meals_{res}$).

$$P_{ill} = \frac{Ill_{res}}{Meals_{res}} \text{ [Equation 4]}$$

As explained earlier, we derive $Meals_{res}$ considering the initial contamination of the intact beef carcass (IB) and through the production of ground beef (GB), not the contamination of the meal as consumed. Food preparation will likely modify pathogen prevalence and load, but such practices are unlikely to change as result of AMU changes, so by using $Meals_{res}$ to calculate P_{ill} we avoid the issue of modeling the risk per prepared meal since surveillance data focuses on production and slaughter.

To provide context, we also calculated P_{meal}, the Ill_{res} per consumption of any meal of beef $Beef_{dtot}$, irrespective of contamination:

$$P_{meal} = \frac{Ill_{res}}{Beef_{dtot}} \text{ [Equation 5]}$$

See Appendix Figure 1.

2. Testing for temporal changes

We tested for monotonic yearly change for all outcomes via Mann-Kendall test for the overall study period, and bootstrapped the test statistic to calculate a of a consistent increase (*4*).

Using numerical integration (*5*), we computed the posterior confidence in pairwise year-to-year differences and in the difference between the mean of the parameter in the last five years versus the remaining years.

3. Scenario analysis: Effect of hypothetical restriction on AMU in beef production

a. Relationship between AMU and antibiotic-resistant non-typhoidal salmonellosis in beef

We used unpublished nationwide data (C.P. Fossler, pers. comm., 2018). from the NAHMS feedlot survey (*6*) to model nontyphoidal salmonella prevalence in cattle RWA vs raised under conventional (CONV) AMU practices: we estimated the sample-positive prevalence

of nontyphoidal salmonella ($Prev_{NTS,CONV}$ and $Prev_{NTS,RWA}$), and the fraction of nontyphoidal salmonella isolates with AMR ($Prev_{AMR,CONV}$ and $Prev_{AMR,RWA}$).

The relative risk (ΔRR) of antibiotic-resistant non-typhoidal salmonellosis prevalence in cattle RWA versus CONV was estimated as:

$$\Delta RR = \left(\frac{Prev_{NTS,RWA}}{Prev_{NTS,CONV}}\right) \times \left(\frac{Prev_{AMR,RWA}}{Prev_{AMR,CONV}}\right)$$ [Equation 6]

b. Prediction of changes in Ill_{res}

We constructed two scenarios to evaluate Ill_{res} changes from a hypothetical AMU restriction in beef production, assuming no changes in consumer habits and food preparation.

We modified the methods described by Williams et al. (*7*) to model the change in Ill_{res} if switching all production to RWA, as follows:

$$\Delta Ill_{res} = Ill_{res} \times (1 - \Delta\, RR)$$ [Equation 7]

where ΔRR is estimated in Equation 6.

In scenario 1, by using ΔRRs rather than prevalence of antibiotic-resistant non-typhoidal salmonellosis, we assumed that animal-level prevalence is proportional (but not equal to) prevalence in meals and ΔRR has a direct linear (i.e. 1:1) effect on ΔIll_{res}.

To relax this assumption, in a second scenario we empirically estimated the relationship between antibiotic-resistant non-typhoidal salmonellosis prevalence in beef and Ill_{res} via Poisson regression, and used it to create an adjustment factor to the calculations done for scenario 1.

In scenario 2, we empirically estimated the relationship between antibiotic-resistant non-typhoidal salmonellosis prevalence in beef and Ill_{res} via Poisson regression (see section i. below). Then, to relax the assumption of scenario 1, we used the slope of this Poisson regression to create an adjustment factor to Equation 7 (Appendix Figures 2- 5).

We tested the confidence in ΔIll_{res} being less than zero (i.e. reduction of human antibiotic-resistant non-typhoidal salmonellosis illnesses) using numerical integration.

i. Regression between Beef nontyphoidal *Salmonella* and -resistant non-typhoidal salmonellosis human illnesses

To adjust the estimated AMR illnesses associated with antibiotic-resistant non-typhoidal salmonellosis prevalence in beef under a 100% raised-without-antibiotic production system, the relationship between prevalence in beef and illnesses may either be assumed to be 1:1 or may be adjusted by an empirically estimated adjustment factor. The adjustment factor was calculated using a Poisson regression of illnesses with beef-attributed antibiotic-resistant non-typhoidal salmonellosis predicted by the centered and transformed product of the prevalence of salmonella and percentage of resistance in beef salmonella as the predictor of case count in one year.

$$\mathrm{Pred}_{trans} = \sin^{-1}\sqrt{Prevalence \times Resistant\ \%} - mean(\sin^{-1}\sqrt{Prevalence \times Resistant\ \%})$$

This regression was carried out omitting the years 2003 and 2009, as these years with abnormally large case counts (Appendix Figure 2) impact prevalence (Appendix Figure 3) rather than resistance (Appendix Figure 4). This is because the relationship between human resistant cases and % resistance is improved by the inclusion of these years, but the regression of prevalence vs human resistant cases is worsened (Appendix Figure 5). The coefficient of the transformed predictor was estimated to be 59.36 (SE 42.3, p=0.233), and the intercept was 6.68 (SE 0.34).

Appendix Table. Variable distributions and parameters used in the computation of the probability of beef-attributable resistant illness per meal made with beef contaminated with resistant nontyphoidal Salmonella

Variable	Definition	Distribution type	2010 Distribution Parameters	2010 Distribution Summary Statistics	Source
NTS_c	Cases per state per year in FoodNet States of Non-Typhoidal Salmonella by Serotype	Discrete	580, 686, 479, 2785, 468, 451, 343, 295, 989, 1063	total cases=8483 national est = 53404	CDC FoodNet
USFN	Multiplier per state per year to scale the catchment area of FoodNet states to the US population	Discrete	0.017, 0.011, 0.031, 0.014, 0.012, 0.007, 0.009, 0.019, 0.021	total % of US population = 14.1%	CDC FoodNet
FB	Fraction of cases of nontyphoidal salmonellosis which are attributed to food	Pert	min=0.91, mode=0.94, max=0.96	mean=0.94, sd=0.01	Scallan et.al 2011.
UD	Underdiagnosis multiplier	Gamma	shape=32.83, scale=0.74	mean=24.3, sd=4.2	Ebel et al., 2016
GBA	Proportion of Foodborne cases attributed to ground beef	Beta	α=1, β=3260	mean=0.0003, sd=0.0003	CDC NORS
WBA	Proportion of Foodborne cases attributed to intact beef	Beta	α=1, β=3260	mean=0.0003, sd=0.0003	CDC NORS
DA	Proportion of cases acquired in the United States	Pert	min=0.07, mode=0.11, max=0.15	mean=0.89, sd=0.015	Scallan et.al 2011.

Variable	Definition	Distribution type	2010 Distribution Parameters	2010 Distribution Summary Statistics	Source
AMR_{perc}	Fraction of nontyphoidal *Salmonella* samples in the NARMS CDC data (matched to NORS outbreaks attributable to beef) with resistance to 1 or more AMD	Beta	α=2, β=1; α=1, β=5; α=0, β=13; α=2, β=1	mean=0.046, sd=0.014	CDC NARMS
GBF	Proportion of beef production sold as ground beef	Discrete	0.58	0.58	NCBA
$Beef_{dtot}$	Total beef disappearance annually, adjusted for food waste in lbs	Discrete	3.91×10^{12}	3.91×10^{12}	USDA ERS
MS_{IB}	Meal Size of beef consumed, not ground, in lbs	Normal	mean = 67.2 grams, sd=3.9	mean = 67.2 grams, sd=3.9	NHANES
Pp_{IB}	Percent Positive for nontyphoidal *Salmonella* from carcass sampling per establishment per year	Beta	for establishment 187, α=5, β=82	mean=0.057. sd=0.025	USDA FSIS
AMR_{IB}	Proportion of nontyphoidal *Salmonella* resistant to AMR from NARMS USDA carcass samples	Beta	α=97, β=152	mean=0.39, sd=0.031	USDA NARMS
MS_{GB}	Meal Size of beef consumed, ground, in lbs	Normal	mean = 61.0 grams, sd=4.8	mean = 61.0 grams, sd=4.8	NHANES
Pp_{GB}	Proportion Positive for nontyphoidal *Salmonella* from ground beef sampling, per establishment per year	Beta	α=34, β=54	mean=0.61, sd=0.05	USDA FSIS
AMR_{GB}	Proportion of nontyphoidal *Salmonella* resistant to AMR from NARMS FDA retail ground beef samples, and by establishment for USDA FSIS samples	Beta	for establishment 795, α=4, β=2	mean=0.67, sd=0.18	FDA NARMS, USDA FSIS
$Prev_{NTS,CONV}$	Prevalence of nontyphoidal *Salmonella* in the NAHMS study among conventionally-raised cattle.	Beta	α=678, β=4940	mean=0.12, sd=0.005	Unpublished data
$Prev_{NTS,RWA}$	Prevalence of nontyphoidal *Salmonella* in the NAHMS study among raised-without-antibiotic cattle.	Beta	α=54, β=679	mean=0.09, sd=0.01	Unpublished data
$Prev_{AMR,CONV}$	Proportion of nontyphoidal *Salmonella* with AMR in the NAHMS study among conventionally-raised cattle.	Beta	α=134, β=650	mean=0.26, sd=0.02.	Unpublished data
$Prev_{AMR,RWA}$	Proportion of nontyphoidal *Salmonella* with AMR in the NAHMS study among raised-without-antibiotic cattle.	Beta	α=11, β=66	mean=0.23, sd=0.06	Unpublished data

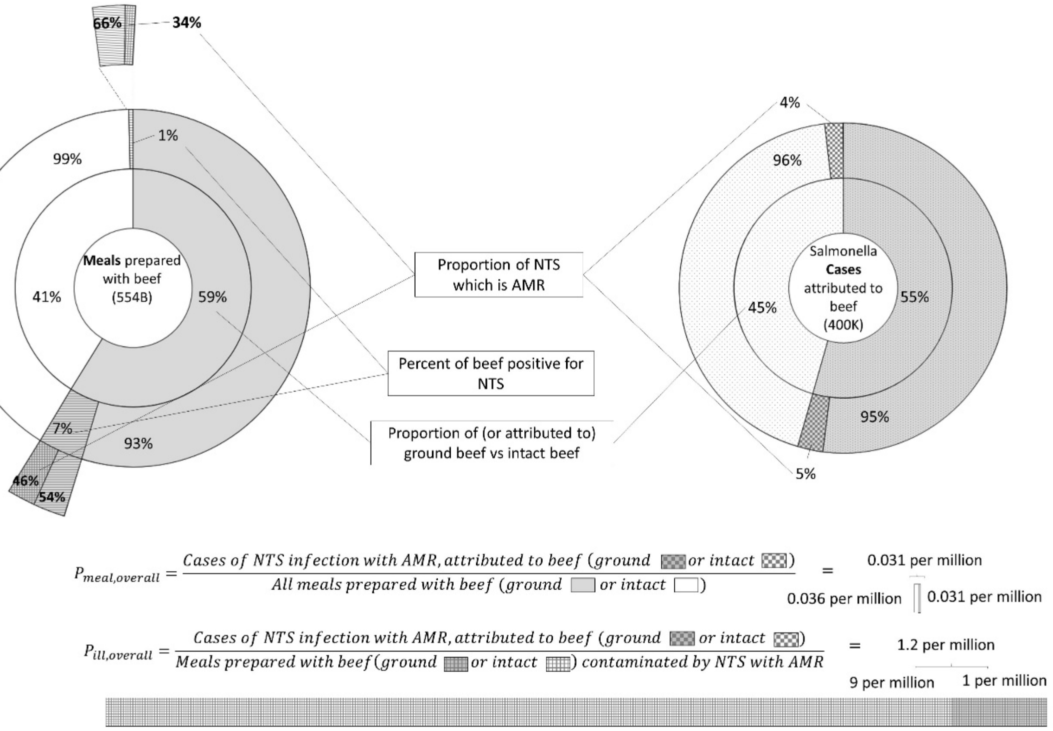

Appendix Figure 1. Probability of non-typhoidal salmonellosis with resistance to one or more antibiotic (antibiotic-resistant non-typhoidal salmonellosis) per million meals made with beef ($P_{meal,overall}$), and per million meals made with beef initially contaminated with antibiotic-resistant non-typhoidal salmonellosis ($P_{ill,overall}$) in 2002-2010. The sunburst diagrams represent the data used for parameterization, stratified by beef type – ground and intact: meals prepared with beef (approximately 554 billion), and human cases of non-typhoidal salmonellosis attributed to beef (over 400,000). The diagrams indicate, from center to periphery: the relative proportions of ground (grey) and intact (white) beef, the proportion of meals contaminated with nontyphoidal Salmonella (stripes), and the proportion of antibiotic-resistant nontyphoidal Salmonella (grid). The symbols in the equations for $P_{meal,overall}$ and $P_{ill,overall}$ refer to the data used for parameterization and represented in the sunburst diagrams, and the bar sizes represent the relative magnitude of these probability means.

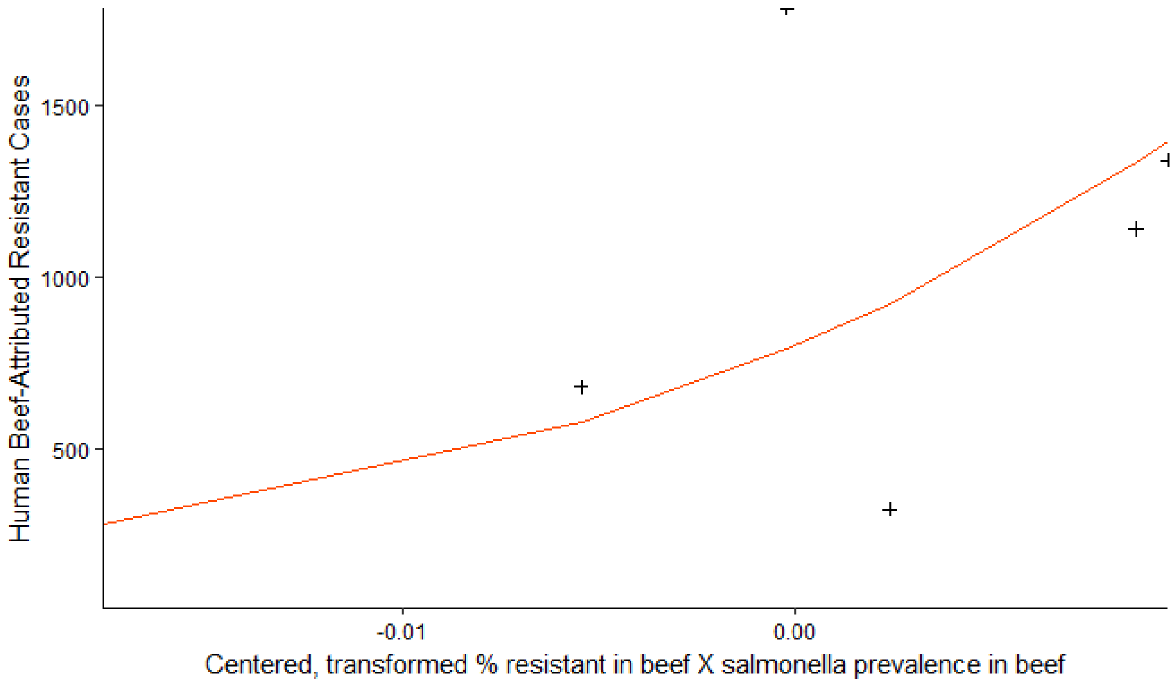

Appendix Figure 2. Poisson regression estimated cases (line) plotted over the human resistant cases and transformed resistance-prevalence in beef.

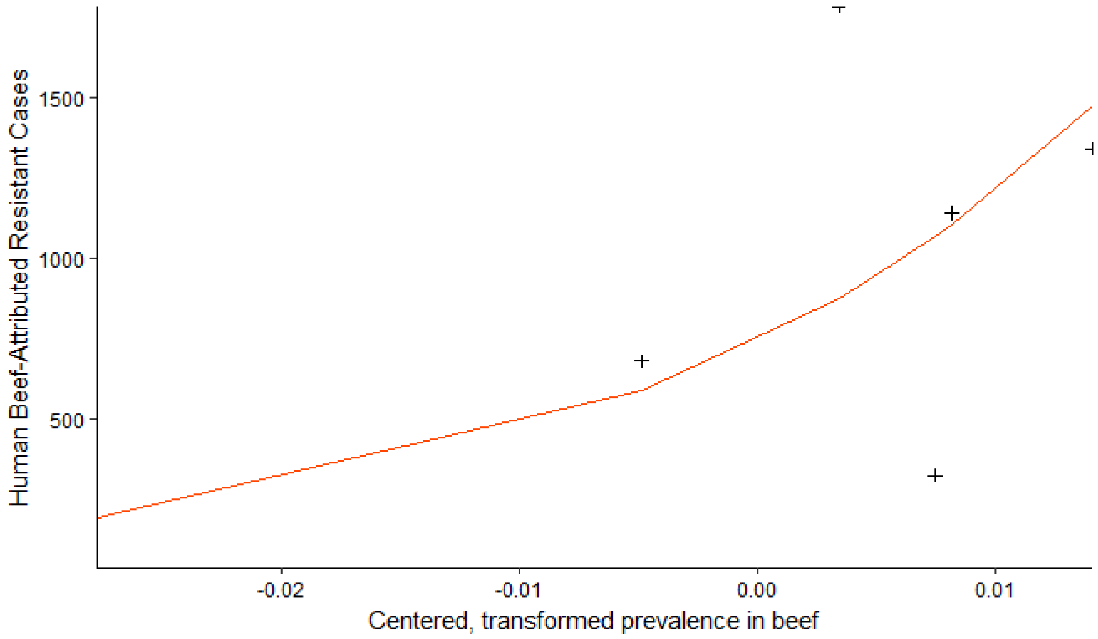

Appendix Figure 3. The centered, transformed prevalence of salmonella in beef as a predictor for human cases with resistance, omitting years 2003 and 2009.

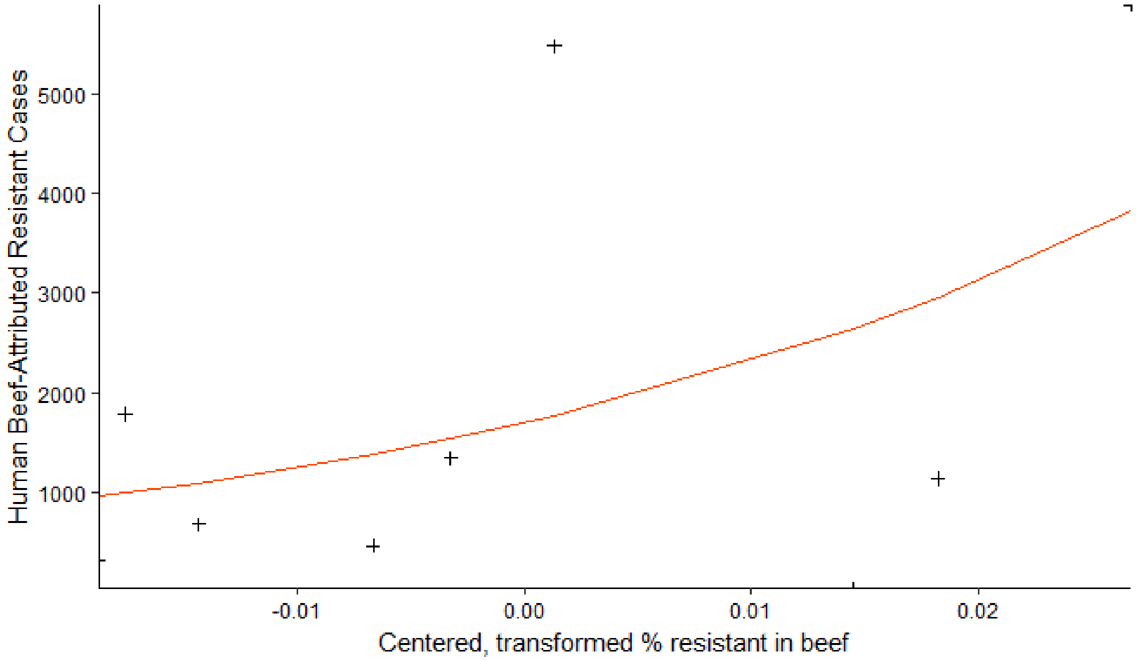

Appendix Figure 4. The centered, transformed percent of beef with resistance as a predictor for human cases with resistance, including all years 2002-2010.

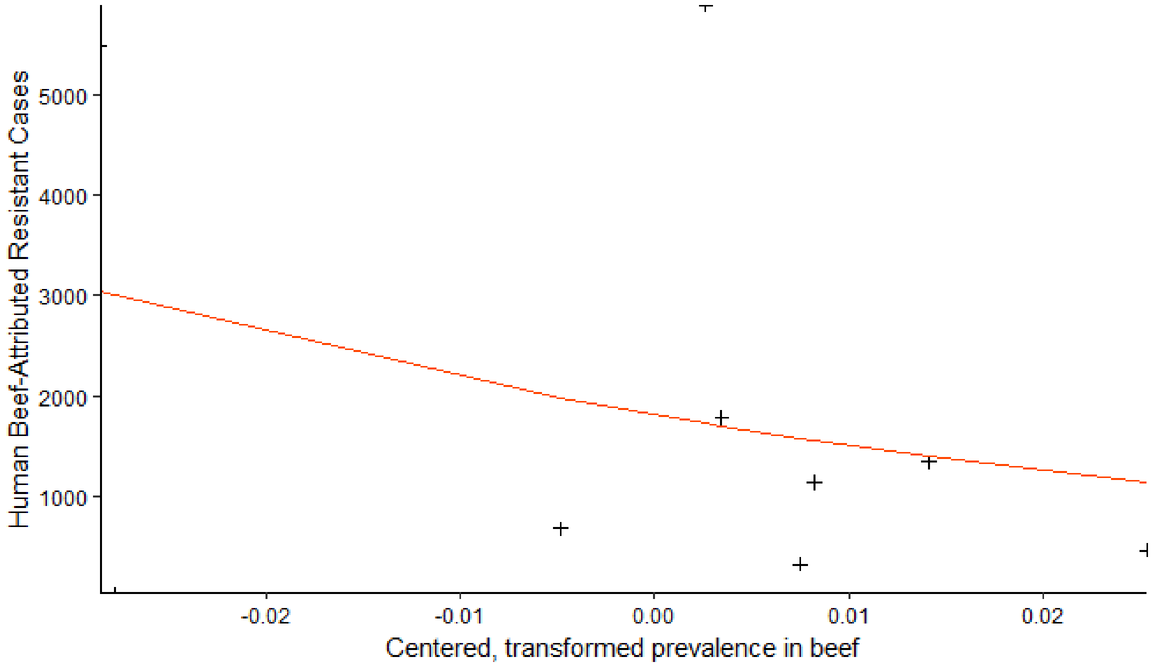

Appendix Figure 5. The centered, transformed prevalence of salmonella in beef as a predictor for human cases with resistance, including all years 2002-2010.

Supplemental Text on Multidrug (MDR) and Clinically relevant resistances (CRR)

The mean $P_{ill,overall}$ for nontyphoidal *Salmonella* $_{MDR}$ was 0.023 per million meals made with beef with nontyphoidal *Salmonella* $_{MDR}$ (95%CrI: 0.011 - 0.1), and beef-attributable nontyphoidal *Salmonella* $_{MDR}$ did not increase over time. The mean $P_{ill,overall}$ for nontyphoidal *Salmonella* $_{CRR}$ was 2.66 per million (95% CrI: 0.006 - 18 per million), and $P_{meal,overall}$ was 0.0268 (0.00011 - 0.12 per million). The mean population incidence of these beef-attributed CRR cases across all years was 0.54 per 100,000 (0.002 - 2.45).

Of the 24 beef-attributable outbreaks in the NORS dataset that were matched to NARMS samples with resistance to any drug, only 6 of those were resistant to just one class. Eleven of the 24 were resistant to 5 or more classes, and most CRR outbreaks fit that part of the definition rather than resistance to fluoroquinolones or third generation cephalosporins specifically. Interestingly, the NARMS samples with Fluoroquinolone resistance which were matched to two beef outbreaks were not resistant to any other antibiotics in the panel. In contrast, the third-generation cephalosporin-resistant outbreaks were also resistant to more than five other antibiotic classes in all but one case – which was resistant to four classes.

The trends in MDR and CRR P_{ill}, $Meals_{res}$, Ill_{res}, and P_{meal} for all cuts of beef combined are shown in Appendix figures 5-8.

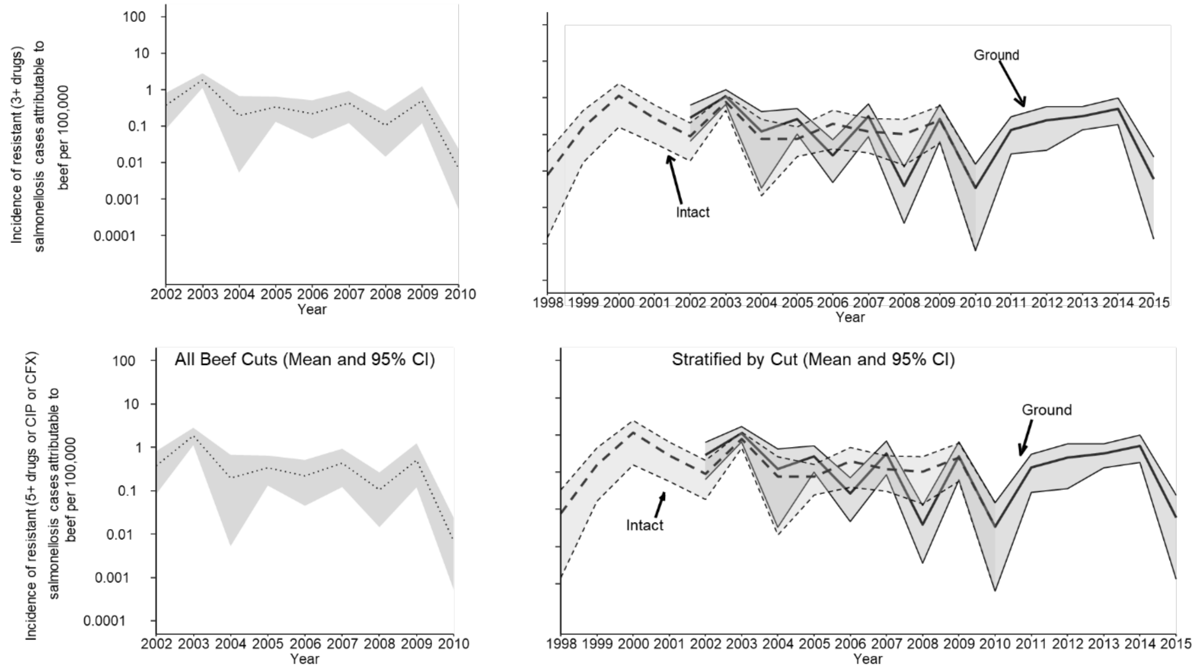

Appendix Figure 6. Mean and 95% Credible Interval of the incidence of salmonellosis cases with resistance to three or more AMD classes, or to more than 4 classes or 3rd Generation Cephalosporins or Fluoroquinolones, attributable to beef (intact, ground, or any) per 100,000 of the US population.

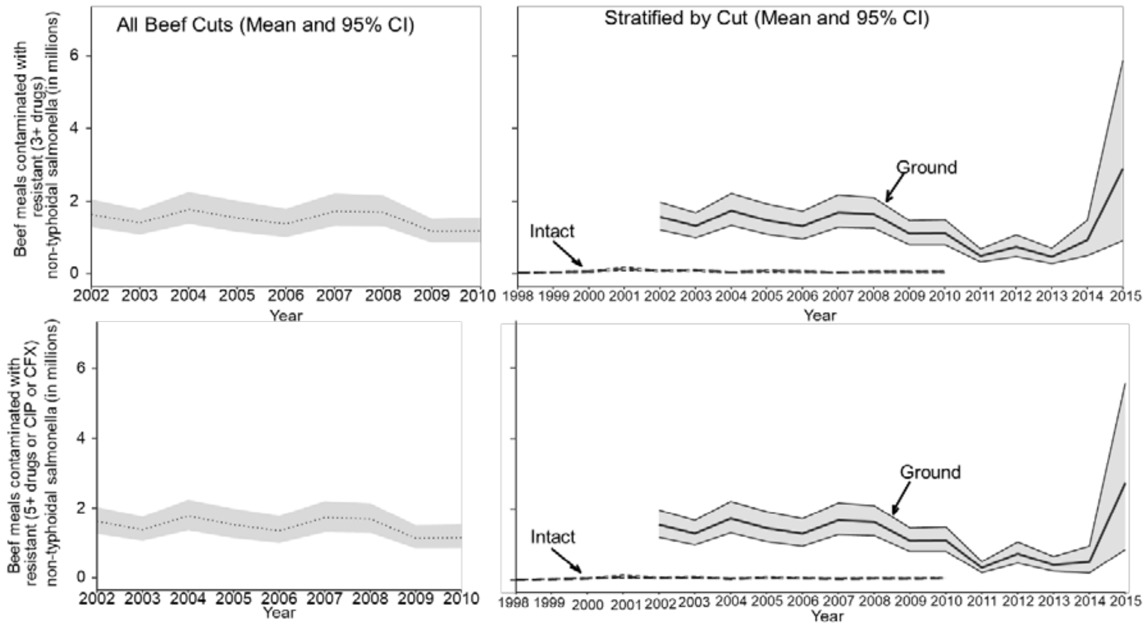

Appendix Figure 7. Mean average estimated consumed beef meals in the millions (ground, intact cuts, or both) with resistance to 3 or more classes (MDR), or to 4 or more classes or 3rd Generation Cephalosporins or Fluoroquinolones (CRR), for all years with available data, with 95% confidence limit.

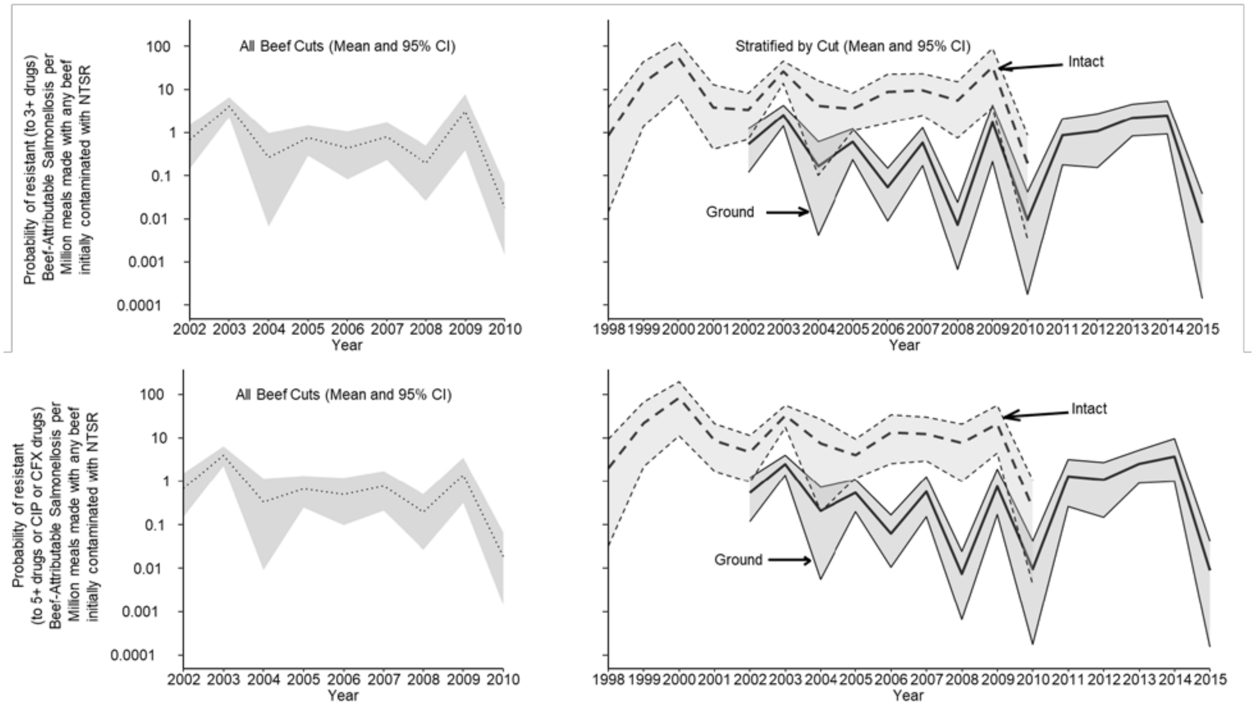

Appendix Figure 8. Mean and 95% Credible Interval of the MDR and CRR P_{ill}, or the Probability of resistant salmonellosis per million meals consumed made with beef containing non-typhoidal salmonella resistant to more than 2 class of antibiotic (MDR) or to more than 4 classes, or specifically to 3rd Generation Cephalosporins and Fluoroquinolones (CRR).

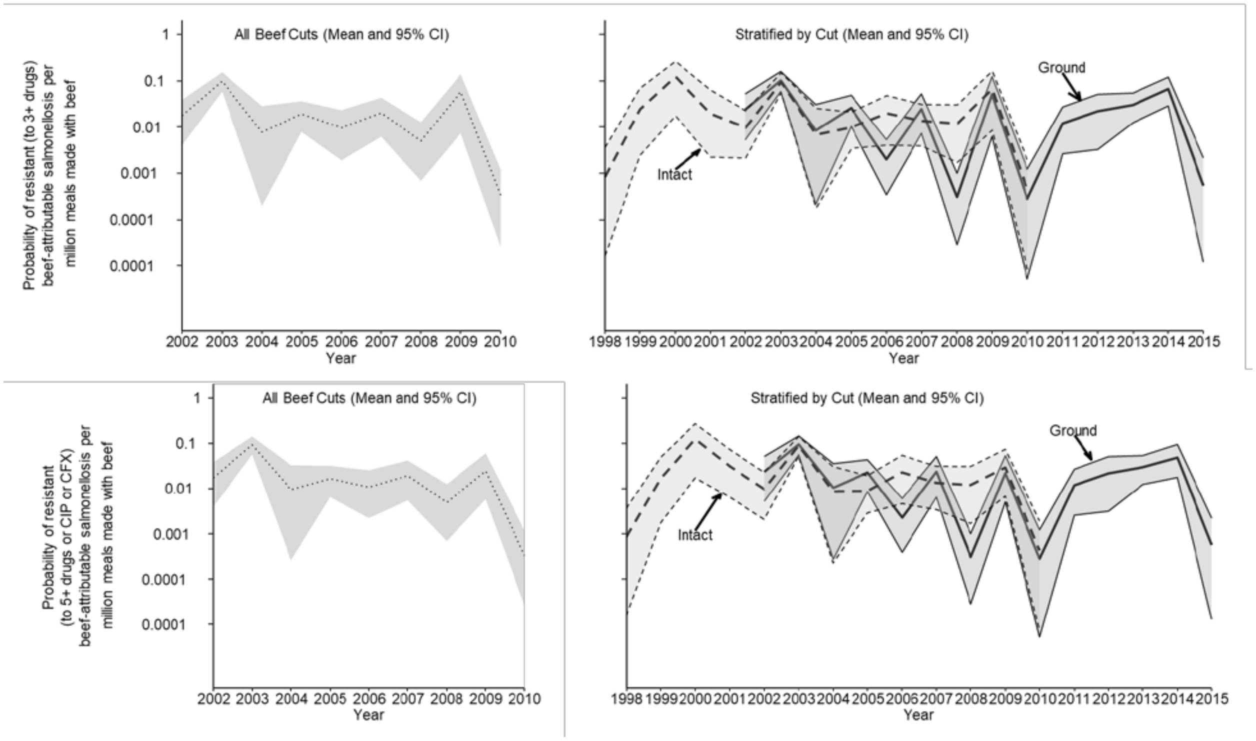

Appendix Figure 9. Mean and 95% Credible Interval of the MDR and CRR P_{meal}, or the Probability of (combined, ground, or intact) beef-attributable salmonellosis resistant to more than 2 class of antibiotic (MDR) or to more than 4 classes, or specifically to 3rd Generation Cephalosporins and Fluoroquinolones (CRR) per consumed meal made with beef per year (irrespective of nontyphoidal *Salmonella* contamination status).

Supplemental Text on Sensitivity Analysis

The most influential drivers of Pill uncertainty were illness attributions for both IB and GB. The fraction of human nontyphoidal *Salmonella*cases which had AMR was third highest (Figure 9). Resistance prevalence in GB was the fourth most influential factor, followed by underdiagnosis and nontyphoidal *Salmonella* prevalence in GB. nontyphoidal *Salmonella* prevalence and resistance prevalence in IB, in contrast, were among the least influential of variables. When considering the change in influence over time, however, nontyphoidal *Salmonella* prevalence in IB steadily increased in importance over the period of the study to become the highest in 2010, (Figure 10). The fraction of nontyphoidal *Salmonella* attributable to IB was the most important factor for every year in the study except 2010, and attribution to GB had the greatest year-to-year change in rank of influence. Uncertainty of AMR prevalence among

human cases and uncertainty in GB nontyphoidal *Salmonella* prevalence remained relatively stable by comparison.

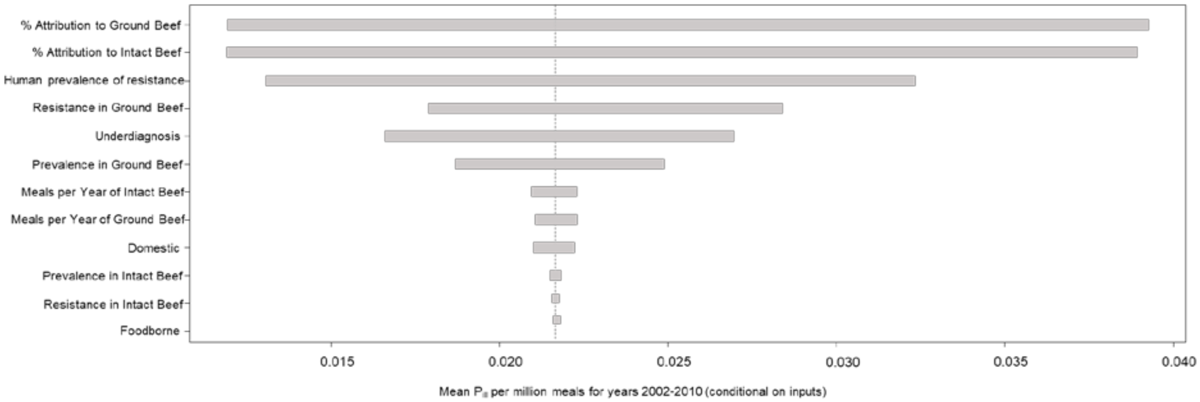

Appendix Figure 10. Tornado plot of conditional means analysis for the average $P_{ill,overall}$ across all 9 years of estimates for all types of beef. The broader the band, the more impact the input variable had in $P_{ill,overall}$.

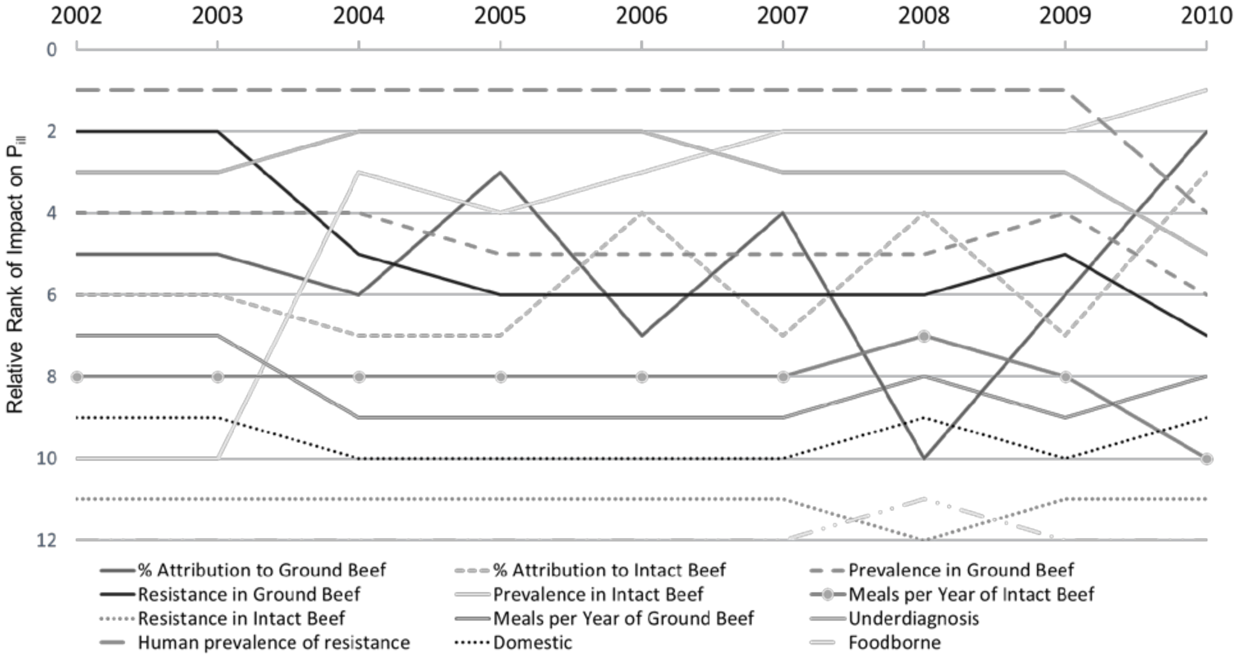

Appendix Figure 11. Yearly rankings of the impact of uncertainty in predictors on the uncertainty of the outcome P_{ill}, where a rank of 1 shows the most impact on uncertainty.

References

1. Centers for Disease Control and Prevention. National Antimicrobial Resistance Monitoring System (NARMS): human data [cited 2018 Oct 18]. https://wwwn.cdc.gov/narmsnow/

2. Centers for Disease Control and Prevention. National Outbreak Reporting System (NORS) [cited 2018 Oct 18]. https://www.cdc.gov/nors/data/using-nors.html

3. Centers for Disease Control and Prevention. National Antimicrobial Resistance Monitoring System for Enteric Bacteria (NARMS) [cited 2019 Mar 18]. https://www.cdc.gov/narms/about/index.html

4. Yue S, Pilon P. A comparison of the power of the *t* test, Mann-Kendall and bootstrap tests for trend detection / Une comparaison de la puissance des tests *t* de Student, de Mann-Kendall et du bootstrap pour la détection de tendance. Hydrol Sci J. 2004;49:21–37. https://doi.org/10.1623/hysj.49.1.21.53996

5. Levy H. Stochastic dominance: investment decision making under uncertainty. 3rd ed. [cited 2020 Feb 12]. https://www.springer.com/gp/book/9783319217079

6. Dargatz DA, Kopral CA, Erdman MM, Fedorka-Cray PJ. Prevalence and antimicrobial resistance of *Salmonella* isolated from cattle feces in United States feedlots in 2011. Foodborne Pathog Dis. 2016;13:483–9. PubMed https://doi.org/10.1089/fpd.2016.2128

7. Williams MS, Ebel ED, Vose D. Framework for microbial food-safety risk assessments amenable to Bayesian modeling. Risk Anal. 2011;31:548–65. PubMed https://doi.org/10.1111/j.1539-6924.2010.01532.x
